# Exploring the General Acceptability and User Experience of a Digital Therapeutic for Cognitive Training in a Singaporean Older Adult Population: Qualitative Study

**DOI:** 10.2196/63568

**Published:** 2025-01-13

**Authors:** Siong Peng Kwek, Qiao Ying Leong, V Vien Lee, Ni Yin Lau, Smrithi Vijayakumar, Wei Ying Ng, Bina Rai, Marlena Natalia Raczkowska, Christopher L Asplund, Alexandria Remus, Dean Ho

**Affiliations:** 1 The Institute for Digital Medicine (WisDM), Yong Loo Lin School of Medicine National University of Singapore Singapore Singapore; 2 The N.1 Institute for Health National University of Singapore Singapore Singapore; 3 Department of Biomedical Engineering, College of Design and Engineering National University of Singapore Singapore Singapore; 4 Division of Social Sciences, Yale-NUS College National University of Singapore Singapore Singapore; 5 Department of Psychology, College of Humanities and Sciences National University of Singapore Singapore Singapore; 6 Yong Loo Lin School of Medicine Heat Resilience & Performance Centre National University of Singapore Singapore Singapore; 7 Department of Pharmacology, Yong Loo Lin School of Medicine National University of Singapore Singapore Singapore; 8 The Bia-Echo Asia Centre for Reproductive Longevity and Equality, Yong Loo Lin School of Medicine National University of Singapore Singapore Singapore; 9 Singapore's Health District @ Queenstown, Yong Loo Lin School of Medicine National University of Singapore Singapore Singapore

**Keywords:** older adults, cognitive training, digital therapeutic, DTx, remote, usability, acceptance, interviews, gerontology, geriatric, elderly, experiences, attitudes, opinions, perceptions, perspectives, interview, cognition, digital health, qualitative, thematic

## Abstract

**Background:**

Singapore’s large aging population poses significant challenges for the health care system in managing cognitive decline, underscoring the importance of identifying and implementing effective interventions. Cognitive training delivered remotely as a digital therapeutic (DTx) may serve as a scalable and accessible approach to overcoming these challenges. While previous studies indicate the potential of cognitive training as a promising solution for managing cognitive decline, understanding the attitudes and experiences of older adults toward using such DTx platforms remains relatively unexplored.

**Objective:**

This study aimed to characterize the general acceptability and user experience of CURATE.DTx, a multitasking-based DTx platform that challenges the cognitive domains of attention, problem-solving, and executive function in the Singaporean older adult population.

**Methods:**

A total of 15 older adult participants (mean age 66.1, SD 3.5 years) were recruited for a 90-minute in-person session. This session included a 30-minute playtest of CURATE.DTx, followed by a 60-minute semistructured interview to understand their overall attitudes, experience, motivation, and views of the intervention. Interviews were audio-recorded and transcribed verbatim, then analyzed using an inductive approach. Thematic analysis was used to identify emerging patterns and insights.

**Results:**

A total of 3 main themes, and their respective subthemes, emerged from the interviews: comprehension, with subthemes of instruction and task comprehension; acceptability, with subthemes of tablet usability, engagement and enjoyment, and attitude and perceived benefits; and facilitators to adoption, with subthemes of framing and aesthetics, motivation recommendations and the role of medical professionals. Our findings revealed that participants encountered some challenges with understanding certain elements of CURATE.DTx. Nevertheless, they were still highly engaged with it, finding the challenge to be enjoyable. Participants also showed a strong awareness of the importance of cognitive training and expressed a keen interest in using CURATE.DTx for this purpose, especially if recommended by medical professionals.

**Conclusions:**

Given the positive engagement and feedback obtained from Singaporean older adults on CURATE.DTx, this study can serve as a basis for future platform iterations and strategies that should be considered during implementation. Future studies should continue implementing an iterative codesign approach to ensure the broader applicability and effectiveness of interventions tailored to this demographic.

## Introduction

As individuals age, cognitive abilities mature at different time points and eventually decline [1]. This age-related cognitive decline commonly affects memory, learning ability, fluid intelligence, and multitasking skills [2,3]. In Singapore, aggregate declines may be particularly significant given its rapidly aging population, whereby nearly 1 in 4 Singaporeans will be over the age of 65 by 2030 [4]. This decline in cognitive abilities not only poses challenges for the workforce but also imposes a heavy burden on the health care system [5]. Given these factors, exploring effective methods to manage the decline of cognitive capabilities within this population would be essential to overcome these challenges.

Research has shown that cognitive training can improve cognitive functioning in older adults and even potentially delay the onset or progression of neurodegenerative disorders such as Alzheimer disease [6,7]. In Singapore, where there is high internet and mobile device penetration [8], cognitive training can be administered as a digital therapeutic (DTx) at scale. DTx are evidence-based software designed to prevent, manage, or treat health conditions [9]. They can be delivered remotely and often incorporate rewards and gamification elements to enhance adherence [10].

We have developed CURATE.DTx, a DTx platform that delivers personalized digital cognitive training by a tablet-ready adaptation of the Multi-Attribute Task Battery (MATB) platform, a flight deck simulator initially developed by the National Aeronautics and Space Administration (NASA) [11] and later further adapted by the United States Air Force (USAF) [12,13]. The MATB platform requires users to simultaneously manage 4 different tasks, which include responding to indicator lights and changing scales, tracking a target using a joystick, following verbal instructions to tune to specific radio frequencies and channels, and managing fuel tank levels. It is a naturalistic multitasking paradigm that has commonly been used as a mental workload measure for different task intensities [14,15] and has strong face validity as, by design, it involves attention, problem-solving, and coordination amongst tasks. Using the platform challenges the executive function by demanding sustained vigilance and goal-directed action for managing multiple tasks while also training attention and problem-solving for each specific task [16]. MATB has also been shown to evoke local and network-level brain responses, characteristic of multitasking [17], and such training may enhance cognitive efficiency and coordination [18].

CURATE.DTx also harnesses CURATE.AI, an adaptive approach that has previously been validated for drug dose optimization in clinical conditions, to personalize a user’s digital cognitive training by correlating difficulty level doses (inputs) to performance outcomes (outputs) using only their own data [19,20]. While this was initially developed for cognitive training in healthy adult and postradiotherapy patient populations, the same application may serve as a potential solution for managing cognitive decline in older adults [19-22].

At its core, CURATE.DTx was designed to retain the primary tasks and functionality of MATB. However, the interface was modernized with gamification, and usability elements were also incorporated into the platform to improve user engagement and overall experience, such as the detailed instructions screens explaining the MATB tasks and a tablet form factor with a touchscreen for intuitive control. The inclusion of these usability features could also improve the accessibility of CURATE.DTx for older adults. Indeed, the widespread adoption of tablets as the medium for game-based interventions for older adults underscores their versatility for this population [23]. In addition, while the use of DTx in older adults is a relatively recent development, initial studies have shown promising outcomes [24,25]. As older adults often have impaired cognitive performance compared to young adults, especially in multitasking-related tasks under high task loads [26], it would be useful for CURATE.DTx to be adapted for this population. Finally, older adults tend to show greater levels of disengagement than young adults when faced with higher task difficulty [27]. Such disengagement could also impact their enjoyment, which is a predictor of motivation when engaging with an intervention [28]. Considering these factors, it is important to understand older adults’ attitudes and experiences with CURATE.DTx, as their feedback is also helpful for improving future iterations of the platform. Therefore, this study aimed to examine the general acceptability and user experience of the CURATE.DTx platform among older Singaporean adults.

## Methods

### Overview

The Consolidated Criteria for Reporting Qualitative Studies (COREQ) guidelines were followed for the reporting of this study [29], with the COREQ checklist presented in Multimedia Appendix 1.

### CURATE.DTx Platform

**Figure 1:**
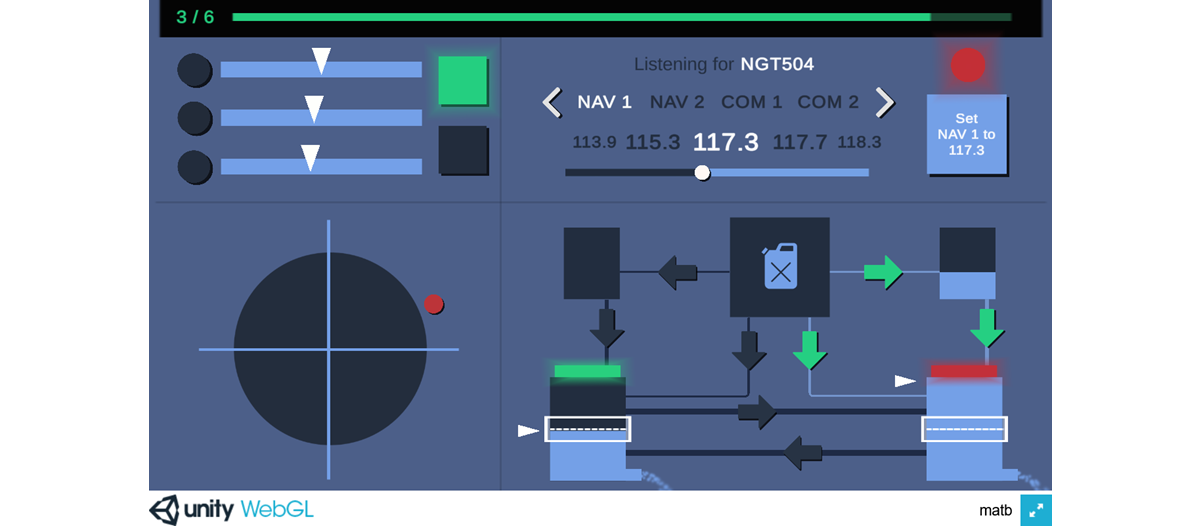
Screenshot of the CURATE.DTx’s user interface. Top left: Systems Monitoring—respond to indicator lights and gauges. Top right: Communications—follow verbal instructions to tune to specific radio frequencies and channels. Bottom left: Tracking—keep the target as close to the center of the crosshair as possible. Bottom right: Resource Management—maintain the fuel tank levels.

The CURATE.DTx platform is comprised of 4 tasks: Systems Monitoring, Communications, Tracking, and Resource Management, as depicted in Figure 1. Systems Monitoring requires the user to monitor the lights and gauges and respond as quickly as possible when they illuminate or change color. Communications requires the user to listen for the call sign “NGT504” and set the radio station and frequency according to the audio instructions, ignoring instructions for other call signs. Tracking requires the user to keep the ball as close to the center of the cross-hair as possible, dragging the ball back when it deviates away. Finally, Resource Management requires the user to maintain the two fuel tank levels within the score area by opening and closing the arrow valves, making appropriate adjustments when the valves occasionally turn off or fail. All 4 tasks can occur at the same time, and the user has to manage them simultaneously until the session ends.

### Recruitment

This study was approved by the National University of Singapore’s institutional review board (reference NUS-IRB-2021-95). Participants were recruited through purposive sampling by age group from a community senior activity center in Singapore. The activity center coordinator identified potential participants, provided an overview of the study, and arranged for informed consent and study sessions. Participants were also told about the research team’s involvement in the study and the reasons for doing the research. The research team (SPK, VVL, SV, and AR) consisted of full-time researchers, 3 female postdoctoral fellows, and 1 male research assistant, and had previous training in qualitative data collection and analyses. They screened potential participants against the following inclusion criteria: (1) Singaporean older adults aged 60 years old and older and (2) fluent in English. Participants with significant hearing, visual, or cognitive impairment that would affect their ability to complete the study were excluded. No relationships with the participants were established before the interview sessions. Recruitment took place over a period of 4 months and ended when the target sample of 15 was obtained. No participants refused to participate or dropped out after consenting to the study.

### Data Collection

This study used semistructured interview sessions to understand the participants’ experiences and attitudes toward CURATE.DTx. Each session was approximately 90 minutes and consisted of a 30-minute playtest where participants play-tested CURATE.DTx on a Samsung Galaxy Tab A7 (SM-T500) 10.4-inch tablet, followed by a 60-minute semistructured interview on their thoughts and experience. To reduce the risk of bias and enhance the credibility and robustness of the results, methods triangulation was used [30]. We compared the participants’ actions observed during their playtest with their verbal qualitative feedback provided in the subsequent interview to ensure consistency of findings across different mediums. All sessions were in-person at the senior activity center’s premises and were conducted in English with at least 2 members of the research team and no nonparticipants present.

Before the session commenced, the research team provided an overview of the study, and informed consent was obtained. All participants consented to the audio recording of the session for transcription purposes, while the participants’ demographic background details were also collected. While performance score data from the sessions were automatically collected, they were not accessed or analyzed as they did not relate to the purpose of this study. Open-ended questions were asked during the interview based on a semistructured interview guide, which was developed to include guiding topics that broadly cover the factors of the Technology Acceptance Model (TAM) framework [31]. The guiding topics used for the interview discussion are presented in Table 1. While the research team’s past familiarity with CURATE.DTx could have influenced them to engage with participants about it in a more positive manner, these guiding topics ensured that both positive and negative feedback were fully captured. Short field notes were also taken where necessary to ensure understanding and clarity of participants’ responses. At the end of the session, all participants were reimbursed for their time. All data collected, including signed consent forms, interview recordings, and demographic data, were stored in a secure database and deidentified for the purpose of this study.

**Table 1:** Semistructured interview topic guide.

Category	Guiding topics for discussion
Interface-specific	Discuss the purpose of the tasks. Discuss understanding of the tasks. Follow up on slips and errors from the playtest session. Discuss the ease of use and difficulties with using the interface.
Program-specific	Discuss the expectations of joining this program. Discuss factors that would increase motivation toward adoption. Discuss barriers and concerns of adoption. Discuss alternatives to this program.

### Data Analysis

The interview recordings were transcribed verbatim, and inductive thematic analysis was used to identify patterns and emerging or recurring themes [32]. Initial coding was conducted on Microsoft Word. Participants’ responses were descriptively labeled and summarized into codes independently by the 4 members of the research team. Any discrepancies in the codes were resolved through discussion. Codes were then organized into potential themes using Microsoft Excel and further reviewed and refined in relation to the entire data set. The final codes and broader themes were generated from multiple discussions and iterations by the research analysis team (SPK, VVL, SV, and AR). Data saturation was achieved when there were no new themes identified and confirmed through discussion within the research team. There were no repeat interviews, transcripts were not returned to participants for comments, and participants did not provide feedback on the findings.

### Ethical Considerations

This study was approved by the National University of Singapore’s institutional review board (NUS-IRB reference: NUS-IRB-2021-95). All participants provided informed consent and were informed of their right to withdraw from the study at any time. All data collected were deidentified for the purpose of this study. At the end of their sessions, participants were reimbursed SGD 45 (US $34) for their time and participation.

## Results

### Participant Characteristics

A total of 15 participants completed the study (10 males, 5 females; mean age 66.1, SD 3.5, range 62-73 years). Participants were from a range of education level backgrounds, with 5 O Levels (10+ years), 6 Diploma (13+ years), and 4 University (16+ years). All participants reported regular use of technology, such as mobile phones, computers, televisions, and tablets. Full participant demographics and characteristics are presented in Table 2.

**Table 2:** Participant demographics and characteristics.

Participant	Age (years)	Gender	Highest education level^a^	Current technology usage
1	63	Male	Diploma	Mobile phone, computer, television
2	67	Male	Diploma	Mobile phone, computer, television
3	67	Male	University	Mobile phone
4	68	Male	O Levels	Mobile phone
5	68	Male	Diploma	Mobile phone, computer, television, tablet
6	67	Male	O Levels	Mobile phone
7	72	Male	Diploma	Mobile phone
8	62	Female	University	Mobile phone
9	66	Male	University	Mobile phone, television, tablet
10	63	Female	Diploma	Mobile phone, television
11	64	Female	University	Mobile phone
12	69	Male	O Levels	Mobile phone, computer, tablet
13	73	Male	O Levels	Mobile phone, computer
14	73	Female	O Levels	Mobile phone
15	64	Female	Diploma	Mobile phone, television, tablet

^a^The years of education based on the Singapore education system is as follows: O Levels=10+ years, Diploma=13+ years, University=16+ years.

### Interview Data

From the participants’ responses, 3 main themes emerged—comprehension, acceptability, and facilitators to adoption. Comprehension describes the participants’ understanding of the CURATE.DTx platform, acceptability covers their thoughts and attitudes towards the CURATE.DTx platform and the tablet hardware, and facilitators to adoption encompass feedback on several recommendations discussed with the participants.

### Theme 1: Comprehension

Under the theme of comprehension, 2 subthemes emerged from participants’ responses, namely instruction comprehension, which is the feedback specific to the “How To Play” instructions page on the web-based platform, and task comprehension, their feedback on the individual MATB tasks and overall gameplay.

#### Instruction Comprehension

Participants initially struggled to understand CURATE.DTx instructions, mainly regarding 2 aspects: the complexity of the instructions and a misconception regarding simultaneous task management.

Participants expressed that the provided instructions were long-winded and difficult to understand, often perceiving them as complicated. Participant 11 mentioned the length of the instructions that, “Firstly, the instructions are very long.” Participant 13 further emphasized their initial struggle, stating, “It is quite complicated actually when you first look at it, I actually struggled initially to try and grasp what this whole thing is about.”

In addition, participants misunderstood the instructions as they did not realize that all tasks had to be played simultaneously. Although it was explicitly mentioned early in the instructions, the sequential presentation of task instructions led some participants to expect CURATE.DTx is to be conducted in a single-task manner. Participant 5 explained this misunderstanding, “The first instruction says four tasks are running at the same time, and it shows during the explanation the four tasks will be coming out, but it is explaining one task at a time, [so I thought] okay so this [CURATE.DTx] is one task at a time.”

#### Task Comprehension

Participants conveyed that it was challenging to understand the more complicated tasks from just the instructions, and that it took some time using the platform to get used to them. As Participant 11 expressed, “I never read instructions fully. I skim. So it's only as I play [that] I more or less get the gist of it, I know what's happening.” However, participants also voiced concerns it might potentially be too complicated and challenging for some in their age group. Participant 9 cautioned, “I guess the elderly, if you give them too many instructions and too many things for multitasking, they might just switch off their mind, they find it a bit difficult for them.”

Participants mentioned that using CURATE.DTx itself helped them understand how the tasks worked. They relied on the in-game task feedback to figure out if they were doing it correctly. Participant 9 remarked, “Initially, I just don’t understand what this is, this whole thing. But after a while, oh yeah, I got the thing.” Participant 5 emphasized the importance of effective task feedback, stating, “In terms of my understanding of the game, the task will give a person better feedback on what to [do], how to react.”

However, participants still expressed a desire for additional assistance and guidance when they faced difficulties in understanding CURATE.DTx. Participant 4 mentioned, “I think like when I’m jammed at a particular part, I ask you all, you all must help me then I [can clearly] understand the game.”

### Theme 2: Acceptability

Under the theme of acceptability, 3 subthemes emerged from participants’ responses. The subthemes included table usability, engagement, and enjoyment of CURATE.DTx, and their attitude and perceived benefits of the intervention.

#### Tablet Usability

Participants mentioned that the usability of the tablet, namely its size and weight, were important factors to consider for the acceptability of CURATE.DTx in this population. While most were satisfied with the physical attributes of the provided tablet, some expressed concerns about other usability aspects of the tablet. Participant 12 shared that, “I think it’s okay. Too big, my age group will have a problem holding. So, lightweight [is better].” However, Participant 10 mentioned the potential issue of screen reflectivity, saying, “Just now the light was glaring, so I have to hold it up so that I can see better.”

#### Engagement and Enjoyment

Participants exhibited high engagement with CURATE.DTx, maintaining a strong level of focus throughout the entire playtest session. Participant 3 remarked afterward, “Like it keeps me on my toes. You didn’t see? If you look at this you will see me very attentive, listening and all this.”

Regarding their enjoyment of various aspects of the DTx, participants acknowledged that certain tasks were more enjoyable than others, with a general preference for the more challenging tasks over the easier ones. Participant 10 stated, “It’s nice to play... Challenging is nice.” Participant 12 had a similar opinion about a simpler task, stating, “This one [Systems Monitoring] is actually very simple, too simple already. Just pressing a button. No fun at all!”

Furthermore, some participants appreciated the presence of a mix of easier and more difficult tasks, as it added variety and enhanced their enjoyment. Participant 13 highlighted this aspect, “In a way, the four [tasks], there is variety... Not all like this [Systems Monitoring], this [is] kind of standard, then I think it's not so enjoyable.”

#### Attitude and Perceived Benefits

Participants expressed that they found CURATE.DTx as an intervention could be potentially beneficial as it was tailored to their prospective cognitive needs, with many considering cognitive training to be important for maintaining their mental capacity in their senior years. Participant 5 emphasized the significance of cognitive stimulation, “For me, my purpose is to stimulate the thinking, the brain, or else your brain deteriorates over time.” Participants viewed CURATE.DTx as a tool for cognitive stimulation and expressed strong intrinsic motivation and interest in using it for cognitive training to challenge themselves. Participant 15 remarked, “I might play this one, for training. Better than the sudoku.”

### Theme 3: Facilitators to Adoption

Under the theme of facilitators to adoption, 3 subthemes emerged from participants’ responses. The subthemes include framing and aesthetics recommendations, motivation recommendations, and the role of medical professionals.

#### Framing and Aesthetics Recommendations

When discussing CURATE.DTx’s visual design, participants suggested that it would be more attractive to older adults if the tasks were framed as real-world, daily life analogies, as they found the current aviation-related tasks to be less relatable. Participant 11 illustrated, “I look at this one, you’ve got petrol, that one is the meter, maybe you can see this car dashboard? And then this one is your air-con slider. And from there at least I can associate, the person also can associate.”

Participants also expressed that the general visual appeal of CURATE.DTx could be improved. They found the color palette to be dull and suggested incorporating more visually appealing elements. Participant 8 elaborated, “If you say that you're going to do it in this color, it is very boring. Because for me, I am a very bright color person, blue, blue, blue? I don’t know.”

#### Motivation Recommendations

When discussing motivation, most participants showed strong intrinsic motivation towards using CURATE.DTx for cognitive training. However, participants also expressed approval for the addition of extrinsic motivation factors, such as the inclusion of a scoring system to CURATE.DTx. They believed that a scoring system would provide them with feedback on their performance and motivate them to strive for improvement. Participant 12 illustrated this viewpoint, saying, “For example, today I score 80, I want to aim for 85. 85, got to aim for 90, 90, 95! People will continue to chase. So if you don't have this, it's [just] another game.”

Some participants thought that CURATE.DTx could be boring over time due to the lack of variety of tasks and levels. As Participant 3 described it, “[it] can be quite boring, to play this everyday. Like there is no changes. Like you see the other games, you see the clearing the blocks game, that one every time change level. That one is different.” They also mentioned that CURATE.DTx lacked some common and attractive features compared to other more attractive existing games in the market. Participant 12 noted, “Most of the time, I play with the music. You see, [the music] attract[s] you, you know?”

The potential gameplay duration of each CURATE.DTx session also emerged as a factor that affected participants’ motivation to use the platform. Overall, most participants were satisfied with the playtest session’s duration, with Participant 12 considering it “not too long, not too short” and Participant 9 saying that, “I think it’s just right.” Participants also emphasized the importance of CURATE.DTx in fitting into their daily schedule. Participant 6 mentioned, “My time is very tight... If I got the time, I don’t mind.” Participant 10 echoed this opinion, saying, “Yes, I would if I have time.”

#### Role of Medical Professionals

When discussing the role of doctors and medical professionals in interventions, most participants expressed a strong belief in the recommendations of their medical personnel, emphasizing that they would heed their advice. Participant 10 succinctly conveyed this sentiment, stating, “Anyone with medical [knowledge] who is looking after me medically, yes, I will take their advice seriously.”

Furthermore, several participants were hopeful that the intervention could serve as a potential alternative to medication. Participant 3 expressed this sentiment, stating, “If this game can help, then why not? Instead of medication.” Since the intervention was in the form of a game, participants generally did not expect any adverse effects. Participant 8 also commented on the absence of adverse effects typically associated with games, stating, “Generally games, the negative effects would be addiction, but I think this one doesn't have.”

Furthermore, participants demonstrated a higher willingness to adopt the platform if it was scientifically proven to be beneficial for their health, even if they wouldn't have considered using it otherwise. Participant 3 elaborated on this point, explaining, “If you say by playing this twice a week, every day, for half an hour, you will improve your mental state of health, then I will definitely play since it is scientifically proven.”

## Discussion

### Principal Findings

This study aimed to understand the acceptability and user experience of older Singaporean adults using CURATE.DTx, a digital cognitive training DTx platform. Overall, we found that participants faced some difficulties with understanding the CURATE.DTx platform. However, engagement with the platform was generally still strong, and participants were also enthusiastic about using it for cognitive training, especially if advised by medical professionals. In light of these findings, there is a need to address several key factors for the next iteration of the CURATE.DTx platform to ensure greater adoption and sustained usage among this older adult demographic group, with these considerations broadly applicable to the development of other similar DTx platforms in this space as well.

### Comprehension Considerations

We identified the importance of considering the comprehension of the instructions on overall experience when developing a cognitive training DTx. Aging has been shown to be associated with declining cognitive abilities that could affect information processing, such as in visual attention [33] and working memory [34]. Specifically, older adults may have a harder time processing unfamiliar visual information and retaining it in memory [35]. We observed this in some of our participants, who struggled to fully understand the game instructions provided on CURATE.DTx and perceived them to be too long-winded and complicated. Some of this difficulty can be attributed to the complexity of several task explanations, as well as the continuous sequential presentation of the instructions. Therefore, older adults would benefit from having complex concepts broken down into smaller portions so that each step is within their capacity for processing, and this information should be summarized again for each task to ensure that it is adequately rehearsed and retained in their memory [36]. Providing accessible bite-sized chunks of information that are reiterated at various points of the instructions will ensure that they are clear and concise, which would prevent misunderstanding and improve their overall experience when using CURATE.DTx. In addition, participants may also benefit from the implementation of instructions explained as an interactive tutorial with the opportunity to practice all 4 tasks simultaneously at the end. This would provide them with the opportunity to familiarize themselves with the tasks and gain a better understanding of the mechanisms involved.

Our findings also showed that providing in-game feedback can be a useful tool for helping older adult users understand the tasks. In line with Nielsen’s usability heuristics, which emphasizes recognition rather than recall [37], incorporating visual elements that provide small reminders of information can aid users in navigating the task without requiring them to remember specific task instructions. Our study participants themselves mentioned a preference for learning CURATE.DTx mechanics through gameplay, using in-game feedback to gauge their performance and adjust their strategies. This is in line with the literature, where feedback in educational games can provide players with schemas to help them learn from their misunderstandings [38]. Indeed, providing regular feedback about their performance enables users to engage in more critical learning [39], and clear and specific feedback—such as indicating correct and incorrect actions, highlighting areas for improvement, and offering suggestions or tips—can direct them to develop more effective game strategies. Furthermore, it is also important to present feedback in a way that is easily understandable to older adults, taking into account their cognitive abilities and visual acuity. Hence, designing a user-friendly interface with intuitive controls, clear visual cues, and accessible feedback would enhance their interaction and learning with CURATE.DTx, thus improving their overall experience.

### Acceptability Considerations

Our findings showed that there was strong interest in using CURATE.DTx for cognitive training. Participants recognized the importance of cognitive training for maintaining cognitive functioning and believed that the CURATE.DTx platform would be useful and beneficial for this purpose. These stated views align with previous research showing that older adults in Singapore are open to adopting technology to achieve their health goals [40]. Furthermore, the intended and perceived benefits of the training platform emerged as the most common facilitators of its use. This finding aligns with the principles of the TAM framework, which suggests that the perceived usefulness of a technological tool strongly influences its acceptance as a learning and training resource [41]. Given the existing intrinsic motivation and expressed interest in CURATE.DTx, ensuring sustained engagement, would thus be key to its long-term adoption in older adults.

Engagement and enjoyment were also found to affect the acceptability of the CURATE.DTx platform. Specifically, participants expressed a preference for more challenging tasks, like Resource Management, which requires them to develop an optimal strategy for balancing resources over simpler tasks, like Systems Monitoring, which merely requires them to click on lights when they illuminate. As facing challenges in a game has previously been reported to directly enhance learning and increase engagement with the game [42], incorporating more challenging tasks can offer participants a sense of accomplishment and keep them motivated. However, we also found that some tasks were reported to be overly complex and difficult to understand for this age group. Thus, striking a “sweet spot” trade-off between challenge and ease of understanding can lead to flow—the sense of engagement and immersion in the interactive experience [43]. While the use of an adaptive training approach such as CURATE.AI can partially aid in this by adjusting the difficulty to an optimal level based on personal performance [19], improving the design of the task elements based on participant feedback would help to ensure a more cohesively enjoyable experience.

The source of the challenge is also an important consideration. While there can be a motivating in-game frustration that causes the player to want to persevere and overcome the challenge, lack of comprehension of CURATE.DTx’s tasks could instead lead to disheartening at-game frustration that causes them to disengage [44,45]. As these seemingly contradictory aspects are rated most highly in terms of importance for older adults [46], successfully finding this balance can make the platform highly appealing to this demographic group.

Finally, participants generally viewed using tablets as the physical medium for CURATE.DTx positively. However, the size and weight of the tablets were highlighted as important factors to consider, underscoring the need for a device that is easy to manage and handle. Despite some concerns about screen reflectivity, the overall design of the tablet was well-received. This is in line with existing literature reporting high satisfaction among older individuals using tablets in clinical settings [47] as well as their perceived benefit not just for cognitive training but also for enjoyment [48]. Given that the effective deployment of a tablet-based DTx in a remote setting use case requires addressing additional factors such as ensuring household internet access, it is important to note that in Singapore, where 99% of resident households, including 93% of senior-only households, have internet access [49], this is less of a concern compared with the digital divide at the global level [50], with disparities in internet access often linked poverty and unemployment [51]. Therefore, understanding the specific needs and circumstances of the target group is crucial for the successful adoption of the tablet-based platform.

### Facilitators to Adoption Considerations

Our findings revealed that enhancing the framing and aesthetics of the current CURATE.DTx platform would make it more appealing and engaging to the older Singaporean population. Specifically, incorporating visually appealing elements and features found in popular games in the current market, such as vibrant colors and attractive visuals, can help to enhance enjoyment. Adhering to design principles, such as judicious color use with no more than 2 or 3 fully saturated intense colors, can assist in accomplishing this [52]. Participants also expressed a preference for tasks framed to be more relatable and familiar to their daily lives, such as a car dashboard, rather than the current MATB flight deck simulator interface incorporated into CURATE.DTx. Again, DTx developers should consider Nielsen’s usability heuristics, specifically the principle of matching the system to the real world [37], to make the platform’s task more intuitive. Using familiar symbols, illustrations, or other analogies that resonate with older adults’ day-to-day lived experiences will ensure that they are able to understand the task requirements more easily. Such aesthetic changes can help to make the platform more appealing to older adults and increase their engagement with the platform [53].

Our findings also highlighted the importance of motivation in the DTx platform design. While participants already demonstrated intrinsic motivation in using the platform, they also welcomed extrinsic motivational factors to further strengthen their interest. Participants were particularly taken with the idea of gamification elements such as a scoring system, reporting that knowledge of their scores would drive them to improve. Enhancing scoring with a leaderboard may be an option to consider in a senior-friendly iteration of CURATE.DTx, as they have previously been shown to motivate players to strive for higher-difficulty goals [54]. Nevertheless, it is important to ensure that extrinsic motivation factors do not undermine participants’ existing intrinsic motivation to use the CURATE.DTx platform but rather enhance it [55].

Participants also supported the introduction of a greater variety of tasks for CURATE.DTx. This can be a means to prevent boredom and also promote meta-learning, a concept of applying knowledge from past experiences to new, related tasks [56]. While the concept is mainly applied in machine learning and artificial intelligence, recent research has proposed that this idea of drawing from past learned experience can be applied in cognitive training, in particular with cognitive control [57]. Promoting such learning could be beneficial to the older adults’ mental health while also increasing the appeal of the CURATE.DTx platform and improving its long-term engagement and adoption.

Finally, our findings revealed the role of medical professionals to be pivotal in the adoption process. In Singapore, health care providers are highly trusted sources for learning about health platforms [58,59], thus playing a critical role in influencing their patient’s decision to adopt new technologies. Participants also highlighted the importance of scientific evidence demonstrating the benefits of the DTx, likely reflecting this population’s trust in research [60]. They also viewed the DTx as a potential alternative to medication, indicating their willingness to explore nonpharmacological approaches to cognitive maintenance. Thus, collaborating with health care professionals and conducting further research studies to demonstrate the efficacy and cognitive benefits of the game could significantly increase trust and promote broader adoption among older Singaporean adults.

### Limitations

Several limitations should be considered when interpreting the findings of this study. First, the study was restricted to English-speaking older adults, which may not fully capture the experiences and perspectives of older adults from non–English-speaking backgrounds. Singapore is a multilingual country, and considering the diverse linguistic landscape, it is important to extend the research to include participants who speak languages other than English. This would provide a more comprehensive understanding of older adults’ acceptability and experiences with the cognitive training digital therapeutics platform.

In addition, it is worth highlighting that this study is focused solely on the particular DTx cognitive training platform used in this research. It’s important to acknowledge that this platform was not originally tailored for older adult users and that moving forward, it is recommended that future iterations be codesigned with the target demographic to develop a more bespoke solution. Future research should explore a wider range of cognitive training interventions and digital therapeutics platforms to gain a more comprehensive understanding of older adults’ acceptability and experiences across different interventions.

### Conclusion

Overall, this study investigated the general acceptability and user experience of a cognitive training DTx in an older adult Singapore population. Despite facing some difficulties in instructional and task comprehension, participants were still highly engaged with the platform and found the challenge provided by the DTx to be enjoyable. Participants also expressed interest and recognized the importance of cognitive training for their population. We also found that certain aesthetics and motivational features and the incorporation of medical professionals into the onboarding process would facilitate greater adoption and long-term usage in this population. These findings highlight several important considerations that can serve as a useful reference to inform the design and implementation strategies of the future iteration of CURATE.DTx as well as other cognitive training interventions for this population.

## Appendix

Multimedia Appendix 1 Consolidated Criteria for Reporting Qualitative Studies (COREQ) checklist.

## Abbreviations

COREQ: Consolidated Criteria for Reporting Qualitative Studies
DTx: digital therapeutics
MATB: Multi-Attribute Task Battery
NASA: National Aeronautics and Space Administration
TAM: Technology Acceptance Model
USAF: United States Air Force
